# In a Quest for Engineering Acidophiles for Biomining Applications: Challenges and Opportunities

**DOI:** 10.3390/genes9020116

**Published:** 2018-02-21

**Authors:** Yosephine Gumulya, Naomi J Boxall, Himel N Khaleque, Ville Santala, Ross P Carlson, Anna H Kaksonen

**Affiliations:** 1 Commonwealth Scientific and Industrial Research Organisation (CSIRO), Floreat WA 6014, Australia; yosephine.gumulya@csiro.au, naomi.boxall@csiro.au, himelnahreen.khaleque@csiro.au, anna.kaksonen@csiro.au; 2 Laboratory of Chemistry and Bioengineering, Tampere University of Technology (TUT), Tampere, 33101, Finland; ville.santala@tut.fi; 3 Department of Chemical and Biological Engineering, Montana State University (MSU), Bozeman, MT 59717, USA; rossc@montana.edu; 4 School of Pathology and Laboratory Medicine, University of Western Australia, Crawley, WA 6009, Australia

**Keywords:** acidophile, bioleaching, biohydrometallurgy, biomining, halophile, metal, microorganism, resistance, tolerance, synthetic biology

## Abstract

Biomining with acidophilic microorganisms has been used at commercial scale for the extraction of metals from various sulfide ores. With metal demand and energy prices on the rise and the concurrent decline in quality and availability of mineral resources, there is an increasing interest in applying biomining technology, in particular for leaching metals from low grade minerals and wastes. However, bioprocessing is often hampered by the presence of inhibitory compounds that originate from complex ores. Synthetic biology could provide tools to improve the tolerance of biomining microbes to various stress factors that are present in biomining environments, which would ultimately increase bioleaching efficiency. This paper reviews the state-of-the-art tools to genetically modify acidophilic biomining microorganisms and the limitations of these tools. The first part of this review discusses resilience pathways that can be engineered in acidophiles to enhance their robustness and tolerance in harsh environments that prevail in bioleaching. The second part of the paper reviews the efforts that have been carried out towards engineering robust microorganisms and developing metabolic modelling tools. Novel synthetic biology tools have the potential to transform the biomining industry and facilitate the extraction of value from ores and wastes that cannot be processed with existing biomining microorganisms.

## 1. Introduction

Biomining is a generic term used to describe the utilisation of microorganisms to process metal-containing ores and concentrates by bioleaching and biooxidation. Bioleaching is typically used in the extraction of base metals, where the metals of interest are solubilised through microbial action and are recovered from solution. Biooxidation is generally used for the pre-treatment of recalcitrant gold and silver bearing minerals, where the microorganisms are used to oxidise the mineral sulfide matrix in which the metal of interest is located. After the undesirable sulfides are dissolved from the minerals, the gold or silver is typically leached with chemical lixiviants, such as cyanide. Both bioleaching and biooxidation utilise similar acidophilic iron and/or sulfur-oxidising microorganisms to solubilise metal containing sulfides.

Biomining can be a feasible alternative for processing low grade ores and ores that contain elevated concentrations of contaminants. Low grade ores can be too expensive to process using traditional methods, and contaminants such as arsenic incur penalties in smelters due to hazardous emissions. Large scale bioleaching has been mainly used for copper, but to some extent also for cobalt, nickel, zinc, and uranium [1]. Moreover, the biooxidation of refractory gold ores has been commercially practiced since 1980s [2]. At present, approximately 15% of copper and 5% of gold are derived from biomining activities worldwide [3]. There are also a growing number of studies exploring the utilisation of biomining to extract metals from various waste streams, such as metallurgical wastes [4] and electronic waste [5].

A number of engineering techniques have been developed to extract metals from minerals through biomining. The most commonly used approaches for commercial scale biomining are based on bioreactors, heaps, and dumps. However, there is increasing interest to use vats and in place or in situ mining for low grade ores [6]. In addition to the extraction of metals, bioreactors with acidophilic iron oxidising microorganisms can also be utilised for the removal of excess iron, sulfate, and other contaminants from hydrometallurgical process waters and the generation/regeneration of biological reagents for use as lixiviants [7,8].

Since the identification of *Acidithiobacillus ferrooxidans* (formerly called *Thiobacillus ferrooxidans*) in the 1940s [9,10], and its well-characterised role in catalysing metal extraction, a large number of other biomining microorganisms have been described and utilised for minerals processing. The types of microorganisms found in various biomining operations may vary depending on the mineral and the process conditions. Examples of acidophilic bacterial biomining genera include *Acidimicrobium*, *Acidiphilium*, *Acidithiobacillus*, *Alicyclobacillus*, *Leptospirillum,* and *Sulfobacillus,* and archaeal genera include *Acidianus*, *Ferroplasma, Metallosphaera,* and *Sulfolobus* [11]. Biomining organisms are able to grow lithotrophically by oxidising ferrous iron and/or elemental sulfur as electron donors to generate ferric iron and sulfuric acid, which attack sulfide minerals. Some biomining microbes can also grow autotrophically using CO_2_ for growth, whereas others are heterotrophic and thus require an organic carbon source [11].

The impurities in low grade complex ores and the scarcity of freshwater in arid areas create challenges for the use of traditional biomining microorganisms as biomining microbes are typically sensitive to high ionic strength and elevated metal concentrations of saline leach liquors. Bioprospecting for novel microorganisms from saline and metal-contaminated environments and adapting them to increasing ionic strengths have to some extent improved the capacity of biomining consortia to resist the stresses associated with these environments [12,13]. However, the idea of using synthetic biology tools for designing and constructing more robust biomining microorganisms is also rapidly gaining interest [14].

At present, no genetically modified organism (GMO) are being used in commercial scale biomining. The use of engineered microbes could provide significant benefits to biomining by increasing tolerance to fluctuating and challenging process conditions, and thus potentially reducing the time required for metal extraction. In addition, there is an increasing wealth of synthetic biology tools becoming available that will drive the development of novel microbes and metabolic pathways for biomining microbes. This paper reviews resilience pathways that can be utilised to enhance the robustness and tolerance of microorganisms in harsh environments, and discusses the challenges and limitations of engineering such microbes for biomining applications.

## 2. Genetic and Microbial Engineering of Biomining Microorganisms

As an emerging research field of bioengineering, synthetic biology has undergone dramatic growth throughout the past decade. Synthetic biology, which can be described as the engineering of biology: the synthesis of complex, biologically based systems, which display functions that do not exist in nature [15], has transformed how scientists can apply biology for the benefit of society. Well-characterised biological parts that are integrated into a well-known host (i.e. chassis) have resulted in many synthetic biology success stories, such as microbial artemisinin production for malaria treatment [16] and automated systems for the construction of DNA-based logic circuits [17]. Given their long history as the biotechnology work-horses, *Escherichia coli* and *Saccharomyces cerevisiae* were the natural choices for synthetic biology chassis. Their convenience of use is driven by the ease of genetic engineering, broadly available genetic tools, and deep knowledge about their biology at the molecular level.

However, many industrially relevant applications involve conditions and substrates that are not suitable for the conventional genetic engineering hosts. When considering biomining as an example, *E. coli* lacks the required key cell characteristics and mechanisms, such as the ability to tolerate multiple stress factors, namely low pH, high temperatures, high metals concentrations and the presence of other inhibitory compounds, which are not currently genetically tractable or feasible to introduce. Therefore, many industrially interesting but previously undomesticated organisms are being increasingly investigated as potential synthetic biology chassis [18,19]. Although the basic design principles of synthetic biology can be applied regardless of the host that is used, the actual engineering at cell level requires well-characterised parts [20] and genetic tools, which are typically very host-dependent. Efficient methods for transformation and genomic integration for knock-out/knock-in, functional selection markers, and inducible expression systems are essential tools for part characterisation. However, part characterisation remains a prerequisite for the design and construction of more complex systems, which in turn may open up new opportunities to overcome technical challenges not solvable with traditional engineering.

Despite a number of complete genome sequence being available for biomining species such as *A. ferrooxidans* [21], *Acidithiobacillus caldus* [22], *Leptospriilum ferriphilum* [23], *Alicyclobacillus acidocaldarius* [24], *Sulfobacillus thermosulfidooxidans* [25], *Ferroplasma acidarmanus* [26], *Metallosphaera sedula* [27], and *Sulfolobus solfataricus* [28], only a handful of genetic modifications have been reported. Some heterologous expression vectors and markerless gene replacements have been developed for biomining organisms (summarised in Table 1), albeit with limited efficiency. Many of these genetic tools can serve as a starting point for establishing platforms for future work on metabolic or microbial engineering of acidophiles.

Initial efforts that are targeted at introducing genes from heterotrophic bacteria into *A. ferrooxidans* was carried out using electrotransformation of shuttle plasmids constructed from *A. ferrooxidans* native plasmid, a pUC18 plasmid and *mer* determinant as a selectable marker [29]. Due to poor efficiency (only 1 out of 30 strains was successfully transformed), an *E. coli* conjugation based method was subsequently developed. The broad range plasmid belonging to incompatibility group Q (IncQ) pJRD215 (with kanamycin and streptomycin as genetic marker) was successfully mobilised to *A. ferrooxidans* with the aid of plasmid RP4 (plasmid incompatibility group P (IncP)) integrated in the chromosome of *E. coli* SM10 [34]. Using the conjugation approach, several proteins have been successfully expressed in *A. ferrooxidans*, namely arsenic resistant genes [35] and rusticyanin [51]. A system for gene knockout in *A. ferrooxidans* has been developed by transferring of a disrupted gene from suicide plasmid to the chromosome by homologous recombination, generating *rec*A gene [46,56] and phosphofructokinase B (*pfk*B) gene mutant [57]. More recently, a reporter system based on β-glucuronidase for studying gene expression in *A. ferrooxidans* has successfully been employed [50]. Similarly, for sulfur oxidising microbes *A. thioooxidans* and *A. caldus*, several efforts in their genetic tools reconstruction have been reported. Small plasmid vectors based on pBBR1MCS-2 (which does not belong to the IncQ or IncP groups) were constructed for expression of *arsABC* operon in *A. caldus*, resulted in a strain that resistant up to 45 mM of NaAsO_2_ [36]. An expression vector of *A. caldus*, constructed from a native plasmid originally isolated from *A. caldus* strain SM-1, was recently developed to express α-ketoglutarate dehydrogenase and succinate dehydrogenase genes in *A. caldus* [37]. A plasmid pSDK-1 (pJRD215 derivatives) containing *E. coli* phosphofructokinase-1 gene was constructed and transferred to *A. thiobacillus*, switching its phenotype from an obligate autotrophic bacteria to heterotrophic growth [58].

Most studied biomining chemolithoautotrophic archaea are *Acidianus brierleyi, Sulfolobus metallicus,* and *Metallosphaera sedula,* mainly due to their ability to tolerate higher metal concentrations as compared to other thermoacidophiles used for mineral sulfide processing [59]. Yet, many of the genetic tools have been reported for obligate heterotroph *Sulfulobus* sp. (e.g. *S. acidocaldarius*, *S. islandicus,* and *S. solfataricus*). This is primarily because these species are the model organisms for studying how archaea survive in extreme environments [60]. *Sulfolobus* can be transformed by electroporation, albeit with low efficiency [33]. To extract the low number of transformed cells from the large background of untransformed ones, efficient selection systems were developed, namely the use of antibiotics with a resistance conferring enzyme or the use of metabolically deficient mutant recipient strains with inactivated genes and complementation of these mutations by using the intact gene variant as selectable marker gene [61]. Only limited number of *Sulfolobus*-*E. coli* shuttle vectors based on antibiotic selection have been described, mainly due to the low stability of antibiotics under the *Sulfolobus* growth conditions. Uracil auxotrophic *Sulfolobus* strains, which contain mutations in their *pyrE* or *pyrF* genes (encode for orotatphosphoribosyl transferase and orotidine-5’-monophosphate decarboxylase, respectively), can be used for selection by providing intact *pyrEF* genes as selectable marker genes in the plasmid [41,43]. Another selection system used mutants with an inactivated copy or a deletion of the *lacS* gene (coding for β-glycosidase), and hence the cells were unable to grow on a medium containing lactose as sole carbon and energy source [43,45]. The first *Sulfolobus*-*E. coli* shuttle vector was developed by Aravalli and Garrett [38] by combining a *Sulfolobus* replicon derived from pGT5 and the *E. coli* vector pUC19, with additional alcohol dehydrogenase cloned as a selectable marker. The second shuttle vector, pEXS-series, is constructed from a part of the genome of the virus SSV1 cloned into pGEM5Zf(-) and contains a thermostabilised *E.coli* hygromycin phosphotransferase as selectable marker [39]. The other shuttle vectors were constructed either based on the virus-plasmid hybrid pSSVx [42] or the cryptic plasmid pRN1 [43]. One of the main challenges in genetic modification of *Sulfolobus* spp. is their limited genetic stability. Mobile genetic elements that are present at very high numbers in *S. solfataricus* can influence the outcome of genetic experiments. *S. acidocaldarius* was shown to have the lowest spontaneous mutation frequency as it does not contain any active insertion sequences [62]. This makes it the preferred host for genetic studies, but *S. acidocaldarius* cannot grow on lactose medium [63] and it contains the restriction enzyme *Sua*I [64], making the selection process and molecular cloning become harder, respectively. A limited genetic tool for chromosomal recombination has been developed for *M.sedula*, based on the approaches performed with *Sulfolobus* sp. Using uracil auxothrophy (a mutant with spontaneous mutation at *pyrE* gene for selection), the gene encoding copper efflux protein *copA* was inactivated, resulted in a knockout strain with compromised metal resistance [65].

Initial expression studies with biomining microbes were focused on the expression of proteins related to their performance in bioleaching. The *mer* operon from *A. ferrooxidans*, consisting of the regulatory gene mercury resistance (*merR)*, and structural genes *merC* and *merA*, was electroporated into mercury ion sensitive strains, resulting in the engineering of mercury resistant variants [29]. Expression of arsenic resistance genes in *A. ferrooxidans* generated strains that can grow on solid medium supplemented with ~20 mM NaAsO_2_, opening the possibility of bioleaching of arsenopyrite-pyrite ores [35]. A recent study has suggested that there is an increasing interest in using chemolithoautotrophic bacteria as an alternative chassis for the production of fuels and chemicals from renewable resources. Heterologous biosynthetic pathways, 2-keto decarboxylase from *Lactococcus lactis* and genes encoding acyl-ACP (acyl-acyl carrier protein) reductase and aldehyde deformylating oxygenase from *Synechoccus elongatus*, have been recently expressed in *A. ferrooxidans* for the production of isobutyric acid and heptadecane, respectively [53].

### 2.1. Genetic Engineering

The combined efforts of genome sequencing and functional genomics have revealed that acidophiles provide a source of unconventional metabolic pathways, such as an acid survival pathway, heavy metal resistance strategies, or Rubisco-free carbon fixation pathway [66,67,68]. Despite the numerous acidophile genomes sequences available (86 genomes of prokaryotic acidophiles were reported in 2015 [69]), achieving a comprehensive understanding of acidophile survival mechanisms is still a standing challenge, primarily due to the high number of lateral gene transfers. This has prevented successful metabolic engineering of acidophiles/neutrophiles for biomining applications.

**Figure 1:**
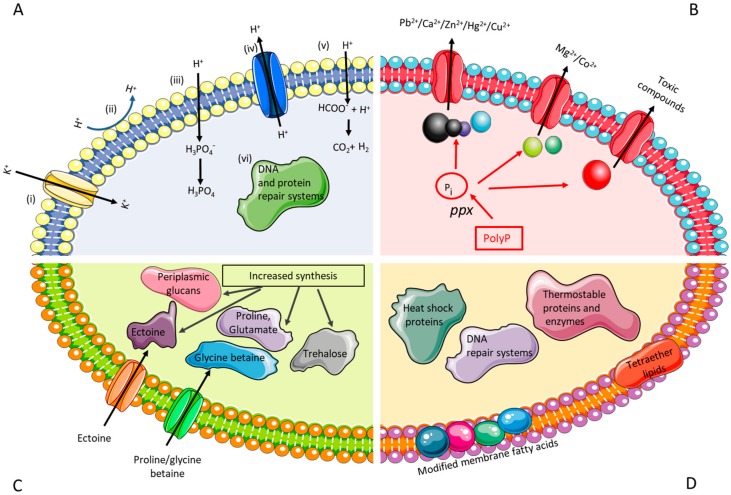
Potential targets for engineering biomining microorganisms: (**A**) **Acid tolerance**. Multiple mechanisms for acid stress tolerance in acidophiles as suggested by Baker-Austin and Dopson [66]: i) Increased influx of potassium into the cell in order to maintain a reversed transmembrane potential, ii) Highly impermeable cell membranes to reduce the influx of protons, iii) Over-production of enzymes/chemicals to bind and sequester protons to maintain pH homeostasis, iv) Increase in active export of protons through transporters, v) Increased synthesis of organic acids to act as uncouplers, vi) Larger proportion of repair systems for DNA and protein repair. (**B**) **Metal tolerance**. Multiple transporters for the efflux of metal cations and toxic compounds to assist in the detoxification of the cell [67]. Additionally, the exopolyphosphatase (ppx) enzyme can convert polyphosphates (PolyP) into inorganic phosphate (P_i_) that will bind to free metal cations and then be transported out of the cell through the transporters. (**C**) **Osmotolerance**. The ability to tolerate high levels of osmotic stress can be achieved through the accumulation of various osmoprotectants, such as ectoine, glycine betaine, trehalose, proline, glutamate, and perisplasmic glucans [73,74,75,76,77,78,79,80,81]. These compounds can either be synthesised in abundance or transported into the cell through transporters when the cell is challenged with osmotic stress. Alternatively, chloride ion channels and pores can be closed to reduce the entry of the ion into the cell [73,74,75,76,77,78,79,80,81]. (**D**) **Thermotolerance**. Incorporation of thermostable enzymes and proteins, increase in DNA repair systems, and expression of heat shock proteins as well as the incorporation of modified membrane composition (fatty acids and tetraether lipids) can help to increase the thermostability of cells [82,83].

The common traits of the biomining microorganisms are that they are acidophilic (survive in low pH environment), autotrophic (capable of deriving energy from the oxidation of inorganic compounds, such as ferrous iron and reduced sulfur compounds) and heavy metal resistant. Several efforts have been conducted in order to characterise the transcriptome and proteome of acidophiles, and these studies have provided insights into differential gene expression governing the various environmental stress responses and their pathways [70,71,72]. However, due to the restrictive access of genetic tools, many of the insights extracted from acidophiles have been verified in *E. coli*. The focus here will be on engineering stress resistance in surrogate hosts (e.g. increasing acid/metal/salt/thermal tolerance of *E. coli*), providing guidelines for future works with biomining microbes. The desired traits of genetically modified biomining microoorganisms are depicted in Figure 1.

#### 2.1.1. Engineering Resistance against Acid Stress

Acidophilic microorganisms have developed several strategies to enable them to thrive at low pH environments. These include: (i) a reversed membrane potential (positive inside) to inhibit the proton (H^+^) entry into the cell via active influx of K^+^; (ii) a highly impermeable cell membrane to reduce the inflow of protons; (iii) producing buffer chemicals to bind and sequester protons; iv) increase in active export of protons through transporters; v) increased synthesis of organic acids to act as uncouplers; and, vi) larger proportion of repair systems for DNA and protein repair (Figure 1A) [66]. Under acid stress, the biomining microbe *A. ferrooxidans* ATCC 23270 tends to expel Na^+^, retain K^+^, and modulate the expression of genes related to cytoplasm pH buffering, namely carbonic anhydrase and polyamine biosynthesis [84]. When grown at pH 1.1, *A. caldus* appears to differentially express transcription factors, suggesting a tight transcriptional control of pH induced genes [85]. Genomic analysis of *A. ferrivorans* showed that the strain has a gene repertoire for low pH adaptation, such as genes encoding for a *kdp*-type potassium uptake ATPase system, hopanoid synthesis, Na^+^:H^+^ antiporter, and arginine and glutamate decarboxylase [86]. Although the physiological role of hopanoids, the sterol equivalents in bacteria, in acid resistance has not been demonstrated, recent studies of deleting squalene-hopene cyclase (the enzyme that converts the linear squalene to cyclic hopene), resulted in low pH sensitive mutant of *Burkholderia cenocepacia* and *Rhodopseudomonas palustris* TIE-1 [87,88]. Bioinformatics analysis of unique core orphan genes of *Acidithiobacillus* spp. suggested that some of them may have functions that are associated with membrane remodelling during cell division as a response to pH stress [89].

The acid stress generated during biomining processes can be harmful to the growth of acidophiles, as demonstrated by the down-regulation of genes related to energy metabolism [65]. Hence, it is very important to increase the acid resistance of biomining microbes, to ensure that the activity of the microbial community and efficiency of the metal leaching processes are maintained. Numerous attempts at engineering acid stress resistance into bacteria have been made with lactic acid bacteria or *E. coli*, mainly for the production of probiotic strains or biofuels, respectively [90,91]. Two strategies have been used. The first aimed at engineering the intracellular microenvironment by engineering amino acid metabolism, introduction of exogenous biosynthetic capacity, and overproduction of stress response proteins. The second strategy was aimed at maintaining cell membrane functionality [90]. Heterologous overexpression of the *Streptococcus thermophilus* decarboxylation pathway in *L. lactis* enabled cells to survive at low pH in the presence of histidine [92]. Introduction of exogenous biosynthetic pathways, such as *E. coli* glutathione synthetase genes, *Listeria monocytogenes* betaine uptake system, and *Propionibacterium freudenreichii* trehalose biosynthetic pathway, increased the survival rate of lactic acid bacteria when they were challenged at low pH [93,94,95]. Heterologous expression of general stress response proteins, namely *E. coli dnaK*, *S. thermophilus shsp, L. casei recO,* significantly enhanced the acid tolerance of *L. lactis* [96,97,98], whereas introduction of *Deinococcus radiodurans* response regulator DR1558 into *E. coli* resulted in a multi-stress tolerant variant [99].

#### 2.1.2. Engineering Resistance against High Metal Concentrations

Many metals are more soluble in low pH than in neutral pH environments, leading to the exposure of acidophiles to high concentrations of metals. Metal resistance can be achieved with the help of a combination of passive (formation of metal sulfate complexes, internal positive membrane potential, competition for binding sites between proton and metals, biofilms/extracellular polymeric substances production) and active systems (efflux pumps/transporters, metal sequestration using inorganic polyphosphates or chaperones, enzymatic conversion of a metal ion to a less toxic form) [67] (Figure 1B). Major protein families that catalyze the transport of metal ions have been identified: P-type ATPases (for Cd^2+^ and Cu^2+^), ATP binding cassette (ABC)-type transporters (for Ni^2+^, Mn^2+^, Fe^2+^, and Mo^2+^), Resistance Nodulation Division (RND) transporters (for Ni^2+^, Co^2+^, Cd^2+^, and Zn^2+^) and cation diffusion facilitator (CDF) family (for Zn^2+^, Cd^2+^, and Co^2+^) [100]. Gene duplication and horizontal gene transfer (demonstrated by the presence of mobile genetic elements) were suggested to play additional roles in the metal resistance of biomining microbes [101]. Recent review related to the resistance mechanisms of biomining bacteria against recalcitrant minerals (e.g. chalcopyrite) is available [59].

With declining ore grades and directing efforts to extract metals from increasingly complex ores that contain gangue minerals with various metallic impurities, biomining microbes are challenged with environments that contain increasing metal concentrations. Engineering biomining microorganisms to tolerate elevated metal concentrations can help to leach low ore grades. Efforts on engineering metal resistant microorganisms have been conducted mainly on mesophilic strains for bioremediation purposes, such as genetically engineered *E. coli* JM109 for bioaccumulation of mercury, nickel, and cadmium by overexpression of a metal transport system and a metallothionein [102,103,104] or a polyphosphate kinase and metallothionein [105]; *E. coli* BL21 for cadmium accumulation by introduction of gluthione synthesis genes, a serine acetyltransferase gene, a phytochelatin synthase gene and a heavy metal ATPase gene [106]; *E. coli* DH5α for adsorption of lead ions by surface display of *Cupriavidus metallidurans* lead binding protein [107]; *Pseudomonas putida* X4 for cadmium biosorption by surface display of metallothionein [108]; and, genetically modified strains of *Corynebacterium glutamicum* for arsenite accumulation (by removing arsenite permease activities required for the release of As^3+^ or overexpression of aquaglyceroporin genes required for improving As^3+^ uptake) [109]. A silver tolerant *E. coli* strain was engineered using a slightly different approach, namely fusing a silver binding peptide AgBP2 to the C-terminus of maltose binding protein, resulting in a silver binding protein with nanomolar affinity for the metal [110].

#### 2.1.3. Engineering Resistance against Salt Stress

One of the most common contaminants in mineral processing is salts, primarily in the form of sulfate or chloride. While sulfate ions in brackish water can introduce osmotic stress to biomining microorganisms, the chloride ions can cause additional damage to the reversed transmembrane potential that acidophiles maintain for survival in low pH environments [75,81]. The chloride ions can additionally interact with counter ions in salt, resulting in the formation of precipitates such as jarosite, which create a passivating layer that prevents further oxidation of the substrate by the microorganisms [75,111]. Typically, the efficacy of bioleaching is reduced in the presence of high salinity, with concentrations as low as 1.5 g/L chloride and 100 g/L sulfate [112]. Some salt-tolerant mixed bioleaching consortia have recently been characterised, which were able to catalyse the extraction of copper from chalcopyrite in the presence of up to 100 g/L sulfate [12,13]. In addition, it has previously been shown that adaptive evolution to increasing sulfate and chloride concentrations can improve salt tolerance of mixed biomining microbial consortia [12]. It appeared that biomining microbes that were classed as moderate thermophiles, able to grow at temperatures between 35 and 50 °C, were the most impacted. Similarly, iron oxidising microbes were more impacted than sulfur oxidising microbes. The generation of ferric iron is essential for the leaching of acid non-soluble metal sulfides, such as pyrite, tungstenite, and molybdenite [113].

The mechanisms used to tolerate salt stress are well studied in neutrophiles and previous studies have shown some of these mechanisms to be counterparts of the salt stress response in biomining microorganisms [75,114] (Figure 1C). Halotolerant microorganisms generally show a biphasic response to osmotic stress, whereby they rapidly uptake potassium ions and accumulate organic acids to compensate for the increasing charge of potassium ions inside the cell [74,75]. This response becomes insufficient at higher levels of osmotic stress, requiring the production of osmoprotectants (low molecular weight compatible solutes), such as ectoine, taurine, hydroxyectoine, trehalose, and periplasmic glucans [73,74]. These osmoprotectants accumulate in the cytoplasm and promote an increase in the osmotic pressure of the cell and subsequently prevent intracellular water loss through osmosis [73,74]. Previous proteomic studies by Zammit et al., [115] and Guo et al., [71] have shown an increase in the abundance of proteins for membrane biosynthesis in acidophiles under chloride stress. This may be a mechanism for repair of damage caused to the cells due to the influx of protons caused by the increased chloride concentrations in the cells. The salt stress response of *A. caldus* showed an increase in proteins for the biosynthesis of amino acids, energy production, and carbon dioxide fixation, suggesting a metabolic shift of carbon flux under salt stress [71]. Studies by Parro et al., [116] have shown that an increase in salinity results in the upregulation of genes for osmo-sensitive potassium channels in *Leptospirillum ferrooxidans*. Furthermore, some thermophiles have also been shown to initially accumulate amino acids, mainly alpha- and beta-glutamate, during the initial influx of potassium ions into cells in response to low-level osmotic stress and then replace amino acids as the osmotic stress increases [73,117].

There have been only few studies on microorganisms that are acid and salt tolerant. This is mainly due to the inability of most iron and sulfur oxidising acidophiles to tolerate high osmotic stress or increased chloride ion concentrations and the limited locations on Earth that provide these environments. *Acidihalobacter prosperus* DSM 5130, *Ac. prosperus* strain F5, *Ac. prosperus* strain V6, and *A. ferrooxidans* strain V8 are among the few halotolerant, iron-, and sulfur-oxidising acidophiles to have their genomes sequenced and are therefore ideal microorganisms for determining the mechanisms used by halophilic acidophiles to tolerate salt stress [118,119,120,121]. All of the genomes were found to contain genes involved in the synthesis of the osmoprotectants ectoine and proline, as well as genes for the synthesis of periplasmic glucans as well as osmolyte transporter [118,119,120,121].

A recent proteomic study comparing the osmotic stress response of the salt sensitive *A. ferrooxidans* ATCC 23370 with that of the salt tolerant *Ac. prosperus* DSM 5130 showed key differences in their response to elevated chloride levels [81]. A generalised stress response with an increase in central carbon metabolism and a decrease in iron oxidation was observed in *A. ferrooxidans* at elevated chloride concentrations. On the other hand, *Ac. prosperus* responded to elevated chloride concentrations by increasing production of the osmoprotectant, ectoine, as well as increasing iron oxidation, potentially due to an increase in the negativity of the Rus protein [81].

Previous studies have shown that the salt tolerance of *Bifidobacterium longum* and *E. coli* can be increased through the increased expression of a small heat shock protein [122] and the *PutP* proline transporter gene [123], respectively. Furthermore, the ectoine genes, *ectABC*, of the halophilic bacterium *Chromohalobacter salexigens* has been successfully engineered into an *E. coli* strain to increase halotolerance [124], while the heterologous expression of a betaine uptake system, BetL has helped to increase the tolerance of *Lactobacillus salivarius* UCC118 to multiple stresses, including osmotic stress [125].

#### 2.1.4. Engineering Resistance against Thermal Stress

The slow oxidation rate of mineral substrates by biomining microbes is one of the main reasons of their limited industrial implementation, especially for chalcopyrite processing [126]. The use of thermophiles can overcome this bottleneck as higher operational temperature can increase the rate of the oxidation, reduce the time for metal solubilisation, eliminate the need for cooling the system, and decrease the passivation of mineral surfaces [127]. Biomining processes above 60 °C usually involve iron and sulfur oxidising archaea, which belong to the genera *Sulfolobus*, *Acidianus*, *Metallosphaera*, and *Sulfurisphaera* [127]. The presence of distinct cell membranes, robust enzymes, and DNA repair mechanism in thermophilic archaea are known to play an important role in their survival in extreme environments (Figure 1D) [82,83,128]. Despite the increasing number of studies involving isolation/characterisation of novel thermoacidophiles, enhancing the thermal robustness of mesophilic biomining microorganisms (those that grow between 20 and 30 °C) could improve leaching efficiency and the potential for more commercial applications of thermophilic bioleaching. Furthermore, there is a growing interest in using thermophiles/extremophiles as chassis organisms or metabolic engineering platforms, namely *Thermoanaerobacter mathranii, Caldicellulosiruptor bescii, S. solfataricus, Thermococcus kodakarensis,* and *Pyrococcus furiosus* [129].

*E. coli,* being the chassis workhorse in synthetic biology, has been engineered, either for enhancing its viability after short heat shock at lethal temperature (~50 °C) or increasing its growth temperature. Heterologous overexpression of exogenous molecular chaperones (αβ-crystallin [130], heat shock proteins (*Oryza sativa* Oshsp16.9 [131], *Oryza sativa* Oshsp90 [132], *Tigriopus japonicas* hsp20 [133], *Caenorhabbditis elegans* Cehsp17 [134], or endogenous transcriptional regulator *evgA* [135] conferred only slight thermotolerance to *E. coli*, suggesting that additional factors are required. Thermophilic microbes that grow effectively at temperatures much higher than 50 °C have been shown to possess membrane lipids with a unique composition or proteins with higher themostability or increased core hydrophobicity [136,137]. Rudolph et al., [138] and Blaby et al., [139] have used adaptive evolution approach to generate a thermophilic descendant (capability of growth at 48.5^°^C or 49.7^°^C) from a mesophilic *E. coli.*

#### 2.1.5. Engineering Iron and/or Sulfur Oxidation Pathways

One of the desirable traits of biomining microorganisms is their ability to gain energy from the oxidation of iron and/or sulfur/reduced inorganic sulfur compounds (RISC). Iron oxidation pathways in biomining microbes have been identified and reviewed exhaustively in [140]. The electron transport chain of *A. ferrooxidans* consists of the outer membrane cytochrome *c* (Cyc2)—where the oxidation of Fe^2+^ to Fe^3+^ occurs, the periplasmic copper protein rusticyanin (RusA), the membrane bound cytochrome *c* (Cyc1), and the integral inner membrane aa_3_ cytochrome oxidase that catalyses O_2_ reduction [141,142]. From rusticyanin, the electrons can take an uphill pathway to the NADH-1 complex, catalysing the reduction of NAD^+^ to NADH, via the membrane bound cytochrome *c* (CycA1), integral cytoplasmic membrane bc1 complex, and the membrane associated ubiquinones [143]. In *A. ferrivorans*, in addition to rusticyanin rusA, Fe^2+^ oxidation can occur through the high potential iron sulfur protein Iro and an isozyme of rusticyanin RusB [144]. In *Leptospirillum* sp, the electron transfer chain is possibly more complex than that in *A. ferrooxidans*, as additional components, such as cytochrome *bd*-quinol oxidase, subunits of cytochrome *c* oxidase, and two predicted bc1 complexes were found [145]. In Euryarchaeal *Ferroplasma*, the oxidation of Fe^2+^ may be mediated by the blue copper protein sulfocyanin and a cbb_3_ terminal electron acceptor [146]. In Crenarchaeota *S. metallicus* and *M. sedula*, a completely different pathway occurs with the presence of cluster fox genes encoding putative cytochromes *b*, heme copper oxidase, ferredoxins, and other proteins with Fe-S binding domain [147,148]. Initial studies of homologous gene expression in *A. ferrooxidans* with genes involved in iron oxidation pathways, namely rusticyanin and cytochrome Cyc2, resulted in an engineered strain with low to moderate improvement in iron oxidation [51,149].

Unravelling the key sulfur oxidation enzymes in biomining organisms appears to be a complicated task as the substrate can exist in various oxidation states and the sulfur oxidation pathway of acidophiles seems to differ substantially from those of most bacteria and archaea. Several enzymes involved in the oxidation of elemental sulfur (S^0^), sulfide, and other RISC have been found in the genome of *A. ferrooxidans*, namely sulfur dioxygenase (oxidises S^0^ to SO_3_^2-^), sulfite:oxidoreductase (oxidises SO_3_^2-^ to SO_4_^2-^), sulfide:quinone oxidoreductase (oxidises S^2-^ to S^0^), thiosulfate quinone oxidoreductase (oxidises S_2_O_3_^2-^ to S_4_O_6_^2-^), rhodanase (disproportionates S_2_O_3_^2^ to S^0^ and SO_3_^2-^), and tetrathionate hydrolase (hydrolyses S_4_O_6_^2-^to S_2_O_3_^2^, S^0^, SO_4_^2-^) (see a detailed review in [141]). The extracellular S^0^ is first transported to the periplasm where it is oxidized by a sulfur dioxygenase to sulfite and by a sulfite oxidoreductase to sulfate. Electrons from S^0^ or RISC are then transferred to oxygen by two respiratory chains, a *bd*-type oxidase or a *ba*_3_-type (or *bo*_3_-type) oxidase via a *bc*_1_ complex. A genome wide microarray transcript profiling analysis of *A. ferrooxidans* grown in S^0^ revealed that cytochrome *bc*_1_ complex (encoded by *petII*) functions in the forward direction by receiving the electrons from the quinol pool and transferring them to either a membrane bound, a soluble cytochrome c, or a high potential iron-sulfur protein, which then gives the electron to the terminal oxidase [150]. Bioinformatic based metabolic reconstruction has predicted new genes involved in RISC oxidation in *A. ferrooxidans*, namely a gene cluster (*rhd, tusA, dsrE, hdrC, hdrB, hdrA, orf2, hdrC, hdrB*) encoding three sulfurtransferases and a heterodisulfide reductase complex (*sat-*encodes an ATP sulfurylase and *sdr*A2 encodes a NADH complex subunit) [151].

The primary challenge in engineering electron transport chain pathway is that many of the most relevant enzymes and electron carriers that are involved in iron and sulfur oxidation are embedded within the cell membrane. Extracellular electron transfer (EET) mechanisms have been studied in detail for *Geobacter sulfurreducens* and *Shewanella oneidensis*, model organisms of microbes that use insoluble electron acceptor (e.g. Fe(III) oxide) and the first successful effort on engineering their extracellular electron transfer chain in *E. coli* has been accomplished by Ajo-Franklin and coworkers [152]. EET mechanisms in microbes that utilised solid state electron donor, such as *A. ferrooxidans* still remains unelucidated as their genetic tools have only been developed recently.

#### 2.1.6. Engineering Carbon Fixation Pathway

Recent attempts to tackle one of the main challenges in synthetic biology—obtaining sustainability in food and energy production via engineering synthetic carbon fixation—have resulted in varying degree of success [153,154,155]. Chemolithoautotrophic biomining microbes employ a wide range of enzymes capable of harvesting reducing power and generating ATP from inorganic sources, thus providing a simpler system for providing sufficient energy required for carbon fixation than the photosynthetic machinery or heterotrophs. Four gene clusters (cbb1-4) in *A. ferrooxidans* genome are predicted to encode enzymes required for carbon assimilation via the conventional Calvin-Benson-Bassham, including form I of ribulose-1,5-biphosphate carboxylase/oxygenase (RubisCO) and the CO_2_ concentrating carboxysomes [68]. Five genes of the carbon fixation cycle of *M. sedula* acetyl/propionyl-CoA carboxylase, malonyl/succinyl-CoA reductase, and malonate semialdehyde reductase) have been successfully expressed heterologously in *P. furiosus*, resulting in a strain that is able to incorporate CO_2_ into 3-hydroxypropionic acid [156].

#### 2.1.7. Metabolic Modelling

Genome-enabled stoichiometric modelling has become a powerful and widely applied systems biology methodology [157,158,159,160,161]. These systems biology techniques construct in silico representations of cellular metabolism based on annotated genome sequences, omics data, physiological data, and literature reviews [162,163]. The metabolic models can organise the large datasets and define possible phenotypes of central metabolism, including electron transport routes, cellular energy production, carbon acquisition, and biomass synthesis. There are two major classes of stoichiometric modelling, which share a common core representation of metabolism: elementary flux mode analysis (EFMA) and flux balance analysis (FBA) [162,163,164]. These stoichiometric modelling approaches apply system wide mass and energy balances to quantify how the system of genome encoded enzymes, along with relevant abiotic reactions, can extract energy from the environment to drive biological processes, including growth and cellular energy production. The approaches do not require difficult to obtain kinetic parameters like V_max_ or K_m_ values to make accurate predictions. More specifically, EFMA identifies all enzymatically distinct and indecomposable flux distributions through a metabolic network; these basic physiological units are termed elementary flux modes (EFMs) [160]. Elementary flux modes, and non-negative linear combinations of EFMs, define all possible steady state metabolisms, making the approach well suited for defining the optimal relationships between electron donors like reduced metals, electron acceptors like oxygen, and biomass or ATP synthesis. Flux balance analysis can identify metabolic routes through the same network models based on linear programming and user defined optimisation criteria, such as maximising growth rate for a culturing environment [163]. These two stoichiometric modelling methods have been applied in many metabolic engineering and ecologically relevant studies including the analysis of flux distributions after gene deletions [165,166,167], selection of heterologous gene expression targets with predictions of phenotypic outcome [157,168,169], identification of optimal genotypes based on criteria such as resource investment into metabolic enzymes or molecular crowding [170,171,172], and quantification of potential mass and energy transfers within microbial populations [161,173,174].

Stoichiometric models have also been built for extremophilic microorganisms and the current section highlights a few examples of thermophiles, halophiles, and acidophiles because of their relevance to biomining. Stoichiometric models of thermophilic microorganisms have been reviewed recently, highlighting features of central metabolism like electron donors and electron acceptor as well as the number of genes and reactions considered in the different models [175]. Many of thermophililc microorganism models focus on lignocellulosic degrading organisms like *Clostridium thermocellum* [176] for biofuel and biochemical production. An exception, relevant to biomining applications, is the hyperthermophilic model archaeon *S. solfataricus* [177], which possess the enzymes to grow chemolithoautotrophically based on sulfur oxidation, bicarbonate fixation, and oxygen respiration. Additional examples of thermomophilic microorganisms not included in the previously mentioned review include a number of Yellowstone National Park hot spring based models, including functional guild-level representations of consortia members from a cyanobacterial mat consortia [174] and *Geoarchaeum* str. OSPB, an archaeal consortia member from an iron oxide mat, which is hypothesised to consume the cellular macromolecules from lysed consortia chemolithoautotrophs [178].

Stoichiometric modelling studies of extremophiles have not been limited to thermophiles but also examined, for instance, halophiles, such as *C. salexigens* [179], *Halobacterium salinarum* [180], and *Halomonas* BC1 and BC2 [181]. The *Halomonas* BC1 and BC2 study integrated genomic data from two brine isolates along with physiological, transcriptomic, and metabolomic data to perform a cellular economics analysis of six common compatible solute synthesis routes. Additionally, mesophilic acidophiles with biomining relevant capabilities like iron or sulfur oxidation have also been modelled as monocultures and cocultures. *A. ferroooxidans* [182,183] has been studied to predict maintenance energy requirements during chemolithoautotrophic growth, while a *A. thiooxidans* study [184] integrated substantial experimental data on sulfur species oxidation with a stoichiometric model to support electron transport pathway reconstructions. Metabolic models of mesophilic acidophile *L. ferriphilum* and *Ferroplasma acidiphilum* cocultures have been examined for potential byproduct cross feeding schemes that could enhance bioleaching [185,186].

### 2.2. Microbial Engineering

Efficient rational engineering of microbial cells requires genetic tools and molecular level knowledge about the metabolic pathways. However, in cases where the molecular level information and tools are lacking, random mutagenesis accompanied with suitable screening or selection method can be utilised to obtain strains with improved properties. For example, classical strain improvement method via mutagenesis has been used for decades as an efficient tool to improve production of industrially-relevant microorganisms [187].

#### 2.2.1. Adaptive Evolution of Biomining Microorganisms

A combined effort of adaptive laboratory evolution (via sequential serial passages in shake flasks or chemostat cultures [188] and mutagenesis) can become a useful approach for evolving biomining microorganisms whose genetic tools are limited. Adaptive laboratory evolution of *M. sedula* resulted in an acid resistant strain (grow well at pH 0.90) with increased copper leaching activity [189]. Over a three-year period *S. solfataricus* was subjected to high temperature serial passage with increasing culture acidity, expanding its limits of thermoacidophily to pH 0.8 and 80 °C [190]. After being cycled for one year (four rounds of increasing elution strength), cells of *Acidithiobacillus* spp. (*A. ferrooxidans* and *A. thiooxidans*) with enhanced adsorption performance, or stronger attachment to the ore particle, were harvested and employed in bioleaching processes, resulting in an improved chalcopyrite bioleaching [191].

As spontaneous mutations occur at a low rate, microbes are often treated with mutagens to increase the frequency of mutations. Artificial mutagenesis, using physical mutagens such as UV radiation or chemical mutagens, such as ethyl methanesulfonate (EMS), can increase the mutation rate up to 100-fold per gene without excessive killing of the cells [192]. EMS mutagenesis of extremely halophilic archaebacteria *Haloferax mediterranei* resulted in a strain with rate of resistance to the antibiotic josamycin increased up to 500-fold [193]. Furthermore, the use of UV induced random mutagenesis is interesting because it is not classified as genetic modification and is hence not susceptible to regulatory procedures.

#### 2.2.2. Engineering Microbial Biomining Consortia

Engineering specialised clonal microbial populations with novel functions and pathways has been clear direction of synthetic biology for the generation of important bio-molecules. In nature, however, microorganisms very rarely occur in individual populations or in isolated environmental niches. Generally, microbial communities are comprised of a diverse range of different microbial species, each with specialised functions that contribute to the evolution, growth, dynamics, stability, and persistence of the microbial community. Natural microbial communities can be difficult to isolate, characterise, and mimic, especially given that a large proportion of environmental microbial species cannot be cultured directly in the laboratory [194]. In addition, the instability of naturally occurring consortia, long growth periods within a laboratory setting, and difficulty in controlling community composition and function can make their use for synthetic biology and biotechnology purposes difficult and sometimes impractical [195,196].

Despite the difficulty associated with the enrichment, growth, and manipulation of natural microbial consortia, there are many reasons for targeting the development of synthetic microbial consortia and engineering microbial communities for synthetic biology purposes, and it has been considered the next frontier in synthetic biology [197,198,199]. Microbial consortia can perform more functions in complicated and variable environments, and are often more tolerant of environmental extremes when compared to monoclonal cultures. The mechanisms by which different species within the microbial community interact, communicate, and co-ordinate their functions and activities still remains largely unknown, but several efforts have been done in controlling ecosystem stability and dynamics [196,200]. In addition, various substrates, compounds, and elements can be converted by individual members of the community, and used by other members for further growth and activity, which allows the functional and metabolic load to be shared across the entire microbial community instead of a single microbial species.

Being able to rationally design synthetic microbial consortia and mimic the conditions and functions required for growth has the potential to improve product yields and culture tolerance to contaminants, with potential application areas of biosensors, biofuels, and bioenergy, wastewater treatment, environmental remediation, and the synthesis of biomolecules for health care and nutrition [194]. As with general synthetic biology concepts, there are two key methods for the development of synthetic microbial consortia, the top down and bottom up approaches. A bottom up approach relies on the rational design of genetic elements, modules, circuits, and pathways to establish metabolic networks and to develop a highly efficient, robust, and controlled microbial consortia. For this to occur, a significant amount of knowledge regarding the diversity, metabolic and physiological functions of the microbial community must be known. The top down approach attempts to re-engineer naturally occurring microbial consortia enriched from a relevant ecological niche [197,201].

For biomining in particular, culture-dependent characterisation of naturally occurring microbial consortia has been difficult and a significant amount of biomining research has been conducted on pure cultures of readily cultivable bioleaching microorganisms, such as *A. ferrooxidans* [21]. However, it is clear from the literature that biomining and associated processes undertaken with microbial consortia consisting of several microbial species that are capable of autotrophic iron and/or sulfur oxidation as well as other secondary functions, such as organic carbon removal, are more efficient than monoclonal cultures, or less diverse microbial communities [113,198,202]. Despite recent advances in detection and characterisation of individual microbial members within biomining mixed culture communities [114,203], enrichment of indigenous microbial communities using culture-dependent methods is still hampered by our inability to mimic the extreme environments in which they exist. In addition, there are still gaps in the complete molecular diversity profile of these microbial communities due to the difficulty in delineating between very closely related species that are often found in these environments.

Groẞkopf and Soyer [195] describe the use of synthetic or defined microbial communities as model systems to overcome the difficulties associated with studying naturally occurring microbial consortia. Much of the biomining microbial consortia research has been conducted with defined communities enriched from these environments. Common biomining microbes such as *A. ferrooxidans, A. caldus, L. ferrooxidans,* and *L. ferriphilium, Ferroplasma,* and others, are often mixed to make defined microbial communities representing those we assume to persist within natural acidic, metal-rich mineral or acid sulfate soils environments. For example, a mixed culture of *A. ferrooxidans* and *A. thiooxidans* was shown to be more efficient at leaching chalcopyrite than a pure culture of either iron or sulfur oxidising microbes, due to the production of additional sulfuric acid, which decreased the formation of jarosite and minimised the passivation of the ore [204]. Similarly, adding a heterotrophic iron-oxidising microbe to a culture enabled the degradation of otherwise inhibiting organic compounds in the leaching environment [205,206]. Other studies have focused on promoting the attachment of biomining microbes to the surface of the mineral and the formation of biofilm, thereby creating a microenvironment which improves the leaching efficiency of the culture [207] and using consortia with higher optimum temperatures to increase the overall leaching kinetics [208]. In addition, partially characterised natural microbial consortia have been enriched and used for various biomining research under stress from contaminants, such as salt [12]. These defined or partially defined microbial communities are exposed to various growth conditions and contaminants to promote tolerance to more extreme conditions that may be encountered in some mineral processes or contaminated sites, with the ultimate goal of enhancing biomining and bioremediation efficiency. A summary of natural and defined mixed microbial consortia used for enhanced biomining and remediation purposes are outlined in Table 2.

Systems biology can be used to systematically understand diverse physiological processes of cells and their interactions and to optimally design synthetic microbial consortia for any given process [211]. Engineering cell-to-cell interactions and communications is at the heart of engineering synthetic communities and optimising biomining of mineral ores [212]. Exopolymeric substances (EPS) play a key role in in biofilm formation [213], and for biomining microbes, the biofilm allows for direct attachment of cells to the mineral surface and the formation of a microenvironment that favours leaching [214]. Several studies have shown that biofilm formation in biomining environments is crucial for interspecies communication [215] and vice versa [21,216,217]. However, further characterisation of the interactions and communication between species within a biomining microbial consortium is required to facilitate engineering new and exciting microbial consortia with novel and industrially relevant functionality.

In addition to engineering the genomes and the interactions and communication between species within a microbial consortium, it is possible to engineer the environment to compliment fine tuning for community composition, activity, and function [197,218]. An example of this was demonstrated by Li et al., [219], whereby biofilm formation was enhanced by modifying one or multiple growth variables to promote the initial attachment of Sulfobacillus thermosulfidooxidans and continuous biofilm development on pyrite. Similar methodologies could be undertaken to fine tune the growth and activity of engineered microbial communities for biomining processes.

Research has been limited to the transformation of pure cultures, including species within the genera Acidithiobacillus and Sulfolobus [51,61]. Generally speaking, the efficiency of transformation for these extremophiles is very low, and further work dedicated to developing methods for the generation of stable transformants, and improving transformation efficiency is required. Brune and Bayer [198] and Rawlings and Johnson [220] both stated that while it could be possible to improve efficiency and yields of bioleaching and biooxidation using engineered microbial consortia, factors such as competition with native microbes, stability of transformed species and engineered communities, process sterility, process conditions, and other regulatory requirements would determine the practicability at industrial-scale. It is likely that maintaining and controlling engineered microbial communities within a non-ideal and non-controlled environment, such as a bioleaching heap or open vat reactor, would be difficult, and the ability to characterise and engineer all complex interactions would be close to impossible. However, as more work is undertaken to fully elucidate the complete microbial diversity in these unique environments and their interactions, the rational design for microbial consortia engineering and overall efficiency of biomining and other associated processes of remediation and waste management could possibly be improved.

## 3. Conclusion

Synthetic and computational biology have the potential to improve the traits of naturally existing microorganisms so they can be productively implemented in biomining and other industrial processes. For acidophiles, the development of genetic tools has lagged behind the developments for other microbes. The delay has not been due to a lack of interest in these microorganisms, but rather a reflection of the difficulties in establishing such a system. In combination with the comprehensive genome-enabled stoichiometric modelling studies, it should be feasible to design genetically engineered microorganisms with higher bioleaching activity, leading to an overall increase in the efficiency of biomining processes. Nevertheless, likewise with the other fields, the applications of GMOs in mining industries would be significantly enhanced by the support of regulatory agencies in developing a safe implementation of the technology.

## Figures and Tables

**Table 1:** Genetic tools for biomining microorganisms.

Organism	*Acidithiobacillus ferrooxidans*	*Acidithiobacillus caldus*	*Sulfolobus* spp. (*S. acidocaldarius*, *S. islandicus*, *S. solfataricus*)
DNA delivery	Electroporation [29], Conjugation [30]	Electroporation [31], Conjugation [32]	Electroporation [33]
Shuttle vectors	pTMZ48, pKMZ51 [29], pJRD215 [34], pSDRA1 [35]	pMSD2 [36], pLAtcE [37]	pAG-series [38], pEXS-series [39], pKMSD48 [40], pMJ03 [41], pMSSVderivatives [42], pA-pN [43]
Selection	HgCl_2_ [29], kanamycin/tetracyline/streptomycin [30,34,35]	Kanamycin/stretomycin [36,37], chloramphenicol acetyltransferase [44]	Hygromycin B [39], Uracil [41,43], lactose [42,43,45]
Markerless gene knockout	Kanamycin mutated allele [46]	Kanamycin mutated allele [47]	Insertion of *lacS* gene [48,49]
Reporter genes	GusA (β-glucuronidase) [50]	-	*lacS* (β-galactosidase) [42]
Regulated gene expression	*tac* promoter [51], *cycA1* and *tusA* promoter [52]	*tac* promoter [36], *tetH* promoter [37]	*aat* promoter [39], *tf55α* promoter [41], *araS* promoter [45]
Protein overexpression	Arsenic resistance genes [35], rusticyanin [51], 2-keto decarboxylase, acyl-ACP reductase, aldehyde deformylating decarbonylase [53]	*arsABC* operon [36],*α*-ketoglutarate dehydrogenase, succinate dehydrogenase [37]	ABCE1 protein [54], IF2 [55]

ACP: Acyl carrier protein.

**Table 2:** Examples of enhanced biomining consortia and their design purposes

Microbial community members	Natural/Defined	Design Purpose	Reference
*Leptospirillum* sp. (MT6), *Acidimicrobium ferrooxidans, Acidithiobacillus caldus, Alicylobacillus* sp. (Y004), *Sulfobacillus* spp., *Ferroplasma* sp. (MT17)	Defined	Reduced jarosite production during chalcopyrite leaching with sulfuric acid produced by sulfur oxidation.	[204]
*A. ferrooxidans* and *Acidophilium acidophilum*	Defined	Heterotrophic removal of inhibiting organic compounds produced during microbial growth.	[206]
*Leptospirillum* MT6 and *A. caldus* and the heterotroph *Ferroplasma* sp. MT17	Defined	Increased acid production.	[205]
*A. ferrooxidans* ATCC 23270, *A. thiooxidans* DSM 622, *L. ferrooxidans* DSM 2391, *L. ferriphilum* DSM 14647 and *A. caldus* S2	Defined	Improved attachment to mineral surfaces. *Leptospirillum* attachment promoted the secondary attachment if *A. caldus* on the surface of pyrite.	[207]
Two strains *A. ferrooxidans* isolated from the coal mine.	Natural isolates	Increased growth and improved leaching rates.	[209]
*A. thiooxidans* A01, *A. ferrooxidans* (CMS), *L. ferriphilum* (YSK), *A. caldus* (S1), *Acidiphilium* spp. (DX1-1), *F*. *thermophilum* (L1), *S. thermosulfidooxidans* (ST)	Defined	Increased growth and improved leaching rates by the introduction of a non-indigenous species to the consortium constructed from indigenous isolates.	[210]
*A. ferrooxidans* ATCC 23270, *A. thiooxidans* (mesophilic) *A. caldus*, *L. ferriphilum* (moderately thermophilic)	Defined	Improved leach yields by promoting growth of moderate thermophiles.	[208]
Uncharacterised environmental salt tolerant, iron and sulfur oxidising enrichment cultures mixed with various mesophilic, moderately thermophilic and thermophilic pure cultures obtained from culture collections.	Mix of natural consortia and defined cultures	Improve salt tolerance with naturally occurring microbes enriched from salty and acidic environments.	[12]
